# Endocrine Disruptor Regulation of MicroRNA Expression in Breast Carcinoma Cells

**DOI:** 10.1371/journal.pone.0032754

**Published:** 2012-03-05

**Authors:** Syreeta L. Tilghman, Melyssa R. Bratton, H. Chris Segar, Elizabeth C. Martin, Lyndsay V. Rhodes, Meng Li, John A. McLachlan, Thomas E. Wiese, Kenneth P. Nephew, Matthew E. Burow

**Affiliations:** 1 Division of Basic Pharmaceutical Sciences, College of Pharmacy, Xavier University of Louisiana, New Orleans, Louisiana, United States of America; 2 Center for Bioenvironmental Research, Tulane University, New Orleans, Louisiana, United States of America; 3 Department of Pharmacology, Tulane University, New Orleans, Louisiana, United States of America; 4 Department of Medicine, Section of Hematology and Medical Oncology, Tulane University, New Orleans, Louisiana, United States of America; 5 Department of Medical Sciences, School of Medicine, Indiana University, Bloomington, Indiana, United States of America; H.Lee Moffitt Cancer Center & Research Institute, United States of America

## Abstract

**Background:**

Several environmental agents termed “endocrine disrupting compounds” or EDCs have been reported to bind and activate the estrogen receptor-α (ER). The EDCs DDT and BPA are ubiquitously present in the environment, and DDT and BPA levels in human blood and adipose tissue are detectable in most if not all women and men. ER-mediated biological responses can be regulated at numerous levels, including expression of coding RNAs (mRNAs) and more recently non-coding RNAs (ncRNAs). Of the ncRNAs, microRNAs have emerged as a target of estrogen signaling. Given the important implications of EDC-regulated ER function, we sought to define the effects of BPA and DDT on microRNA regulation and expression levels in estrogen-responsive human breast cancer cells.

**Methodology/Principal Findings:**

To investigate the cellular effects of DDT and BPA, we used the human MCF-7 breast cancer cell line, which is ER (+) and hormone sensitive. Our results show that DDT and BPA potentiate ER transcriptional activity, resulting in an increased expression of receptor target genes, including *progesterone receptor*, *bcl-2*, and *trefoil factor 1*. Interestingly, a differential increase in expression of Jun and Fas by BPA but not DDT or estrogen was observed. In addition to ER responsive mRNAs, we investigated the ability of DDT and BPA to alter the miRNA profiles in MCF-7 cells. While the EDCs and estrogen similarly altered the expression of multiple microRNAs in MCF-7 cells, including miR-21, differential patterns of microRNA expression were induced by DDT and BPA compared to estrogen.

**Conclusions/Significance:**

We have shown, for the first time, that BPA and DDT, two well known EDCs, alter the expression profiles of microRNA in MCF-7 breast cancer cells. A better understanding of the molecular mechanisms of these compounds could provide important insight into the role of EDCs in human disease, including breast cancer.

## Introduction

Organochlorine pesticides and plasticizing agents are ubiquitous environmental endocrine disrupting compounds with the potential to negatively impact human health [1]. We along with others have demonstrated that the hormonal (estrogenic) activity of these compounds, including o,p′-dichlorodiphenyltrichloroethane (DDT) and bisphenol A (BPA), is mediated through direct binding to the estrogen receptor α (ERα) and subsequent receptor activation [2], [3]. Furthermore, the ER-mediated mechanisms of “environmental estrogens” include both ER-dependent and -independent regulation of gene expression and cell signaling [3], [4], and recent studies have shown that nuclear hormone receptors (NHRs), including androgen receptor (AR), luteinizing hormone receptor (LHR) and thyroid hormone receptor (THR), represent additional targets of endocrine disruptors [5]–[7]. Endocrine disruptors function through targeted agonistic or antagonistic interactions with NHRs, ultimately leading to changes in gene expression. Although the majority of NHR- functional effects are mediated by changes in mRNA transcription, recent studies have demonstrated regulation of microRNA expression as an important mechanism in transcriptome networks.

MicroRNAs, a specific class of endogenous non-coding RNAs highly conserved across species, are single-stranded RNAs of 21–25 nucleotides in length [8]. MicroRNAs regulate mRNA stability and translation by targeting the 3′UTR of target mRNAs and inducing translational silencing [9]. More than 700 microRNAs have been identified in the human genome, and over one-third of all human genes are potentially regulated by microRNAs [10]. MicroRNAs have been associated with many biological processes and disease states [11], [12], including cancer [13]. An important role for microRNAs in hormone signaling has recently been described [14], including estrogen regulation of microRNA expression in the hormone responsive MCF-7 breast cancer cells [15]–[17], and these studies further demonstrate that direct targets of ERα include miR-206, miR-155, miR-125b, miR-145 and miR-21.

miR-21 plays a role in many cell systems, ranging from cardiac hypertrophy [18], [19], to haematopoietic lineage differentiation [20], and oncogenesis [21]–[26]. Estrogen regulation of miR-21 expression has been reported and appears to be complex. Compared to normal breast specimens, miR-21 is overexpressed in the vast majority of breast tumor specimens [27], suggesting that it may potentially act as an oncogene. While inhibition of miR-21 expression has been associated with increased cell growth in cancer [28], Si et al. demonstrated that inhibition of miR-21 by anti-miR-21 oligonucleotides suppressed both cell growth *in vitro* and tumor growth *in vivo* [29]. Estrogen-induced repression of miR-21in MCF-7 breast cancer cells was inhibited by the antiestrogens tamoxifen and fulvestrant, indicating that miR-21 is a direct target of ERα [17]. These data prompted miR-21 to be labeled an ‘oncomiR,’ and miR-21 was found to be significantly higher in ER positive tumors compared to ER negative tumors [30]. Furthermore, an estrogen-mediated decrease in miR-21 was correlated with increased expression of the miR-21 targets PDCD4, PTEN and Bcl-2 at the protein level, which taken together identifies miR-21 as an E_2_-ER-regulated microRNA in MCF-7 cells [17].

Previous reports of estrogen-mediated regulation of microRNAs suggest that endocrine disrupting compounds (EDCs) may also influence microRNA expression. In support of this possibility, Hsu et al. demonstrated that both estrogen and the xenoestrogen diethylstilbestrol (DES) induced miR-9-3 expression in mammosphere-derived epithelial cells, but to our knowledge, this is the only report of EDC-regulation of microRNAs [31]. As EDCs can exert a powerful influence on gene expression networks, we sought to investigate a role for organochlorines and polycarbonates in the regulation of microRNA expression. We utilized classic estrogen responsive reporter gene assays and mRNA analysis of the ER positive MCF-7 breast cancer cell line to initially examine specific transcriptional changes induced my E_2_, BPA, and DDT, and we subsequently used microRNA microarrays to identify EDC-mediated changes in microRNA profiles. Our data show that miR-21 was consistently downregulated by both EDCs tested. Validation of our microRNA microarray findings in MCF-7 cells further suggests that miR-21 expression is mediated by ERα and is a potential regulator of estrogen- and EDC-induced gene expression.

## Results

### Estrogen, BPA and DDT induce ERE transcriptional activity in MCF-7 cells

**Figure 1:**
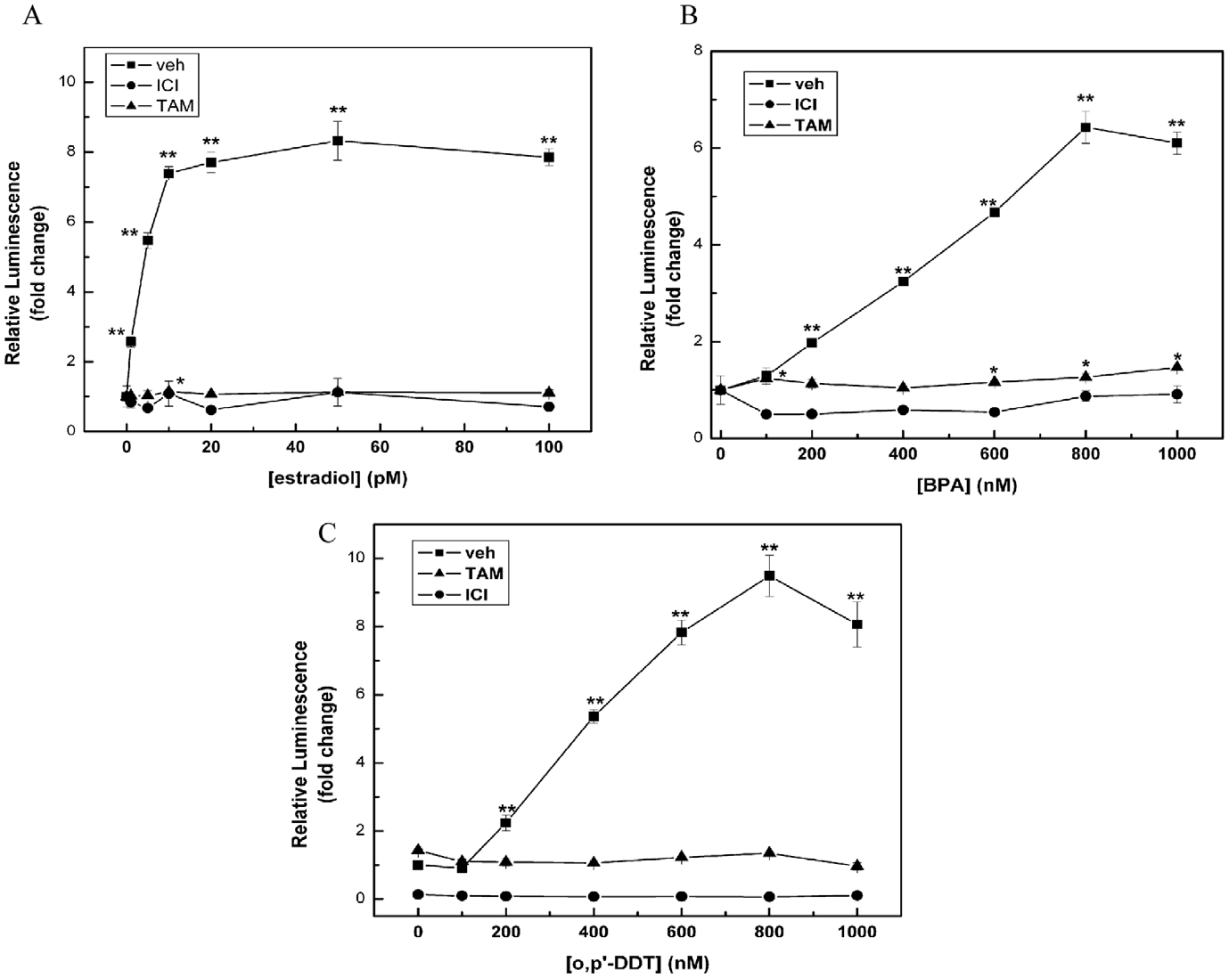
ERE-luciferase assay in MCF-7 cells. Cells were incubated overnight in media containing 5% charcoal-stripped FBS and were then transfected with an ERE-luciferase plasmid. After 4 hrs, drugs were added to the cells as indicated and luciferase levels were measured 18 hrs. later.

To compare overall ERα transcriptional activity of two EDCs (DDT and BPA) with E_2_ treatment, ERE reporter assays in MCF-7 cells were performed. Results indicated an 8-fold increase in the activity of ERα in cells treated with ≥50 pM E_2_ (Figure 1A), in agreement with our and other groups previous studies [32], [33]. Interestingly, DDT and BPA both induced transcriptional activity of the ER; however, a full transcriptional response (i.e., comparable to E_2_) required higher concentrations of both agents compared to estrogen (Figure 1B and 1C). The E_2_, DDT, and BPA-induced transcriptional activity was suppressed by fulvestrant and tamoxifen with all treatments observed, as expected.

### BPA and DDT alter expression of estrogen responsive genes

**Figure 2:**
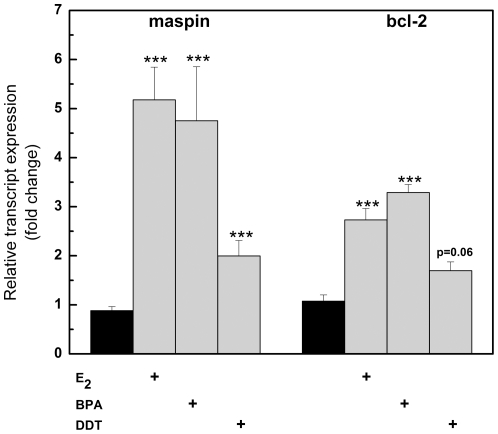
Quantitative PCR confirmation of select genes altered by estrogen, BPA, and DDT in MCF-7 cells. MCF-7 cells were treated with drug as indicated and total RNA was isolated, reverse-transcribed into cDNA, and subjected to real-time RT-PCR analysis for quantitation. Results are shown for (A) SERPINB5 (maspin) and (B) Bcl2 gene expression. Treatment of MCF-7 cells was as follows: Ethanol (vehicle), 1 nM E_2_, 10 µM BPA, and 10 µM DDT. Results are expressed as the mean fold induction ± S.E.M. (***, *p*<0.001; **, *p* = 0.01 – 0.001; *, *p* = 0.05 - 0.01).

Although similarities between EDCs and E_2_ have been described [34], the extent to which BPA and DDT mimic the estrogen profile of select estrogen responsive genes is unclear. To examine the regulation of specific ERα responsive genes by BPA and DDT, we used a PCR array approach and identified genes commonly altered by the three ERα ligands including PgR (211-, 166-, and 172-fold induction by BPA, DDT, and E_2_ respectively), bcl-2 (2.7-, 2.9- and 2.7-fold induction by BPA, DDT, and E_2_, respectively), and other well-known estrogen responsive genes TFF1 (pS2), SERPINB5 and cathepsin D (Table 1). In addition, GABA and gelsolin were downregulated by the EDCs and E_2_ (Table 1). Analysis of two genes, bcl-2 and SERPINB5, by qRT-PCR yielded similar results compared to the array (Figure 2), further validating the estrogenic activity of both DDT and BPA. Interestingly, several genes were differentially regulated by the three compounds. For example, Jun and Fas were increased by approximately 1.8 and 1.5 fold by BPA but were relatively unchanged by E_2_ and DDT (Table 1). These subtle changes in gene expression mediated by the individual compounds prompted us to investigate whether EDC's might alter the microRNA expression profile in MCF-7 breast cancer cells in a manner distinct from that seen by estrogen.

**Table 1:** SuperArray Analysis of Genes Altered by E_2_, BPA and DDT Treatment.

Gene Name	Gene Symbol	E_2_	BPA	DDT
B-cell CLL/lymphoma 2	BCL2	2.68	2.93	0.93
Cathepsin D	CTSD	2.53	2.68	2.41
Gamma-aminobutyric acid (GABA) A receptor, pi	GABRP	−6.83	−5.39	−3.36
Gelsolin (amyloidosis, Finnish type)	GSN	−2.45	−2.76	−3.12
Progesterone receptor	PGR	172	211	166
Serpin peptidase inhibitor, clade B (ovalbumin), member 5	SERPINB5	5.81	4.44	3.75
Trefoil factor 1	TFF1	34	22	23
Jun	JUN	1.09	1.88	1.17
Fas	FAS	1.40	1.54	1.15

Gene expression profile of select genes from a SuperArray analysis showing significant changes in expression with E_2_, BPA or DDT treatment. Numbers indicate significant –fold changes in gene expression.

### E_2_, BPA and DDT alter patterns of miR-21 expression

**Figure 3:**
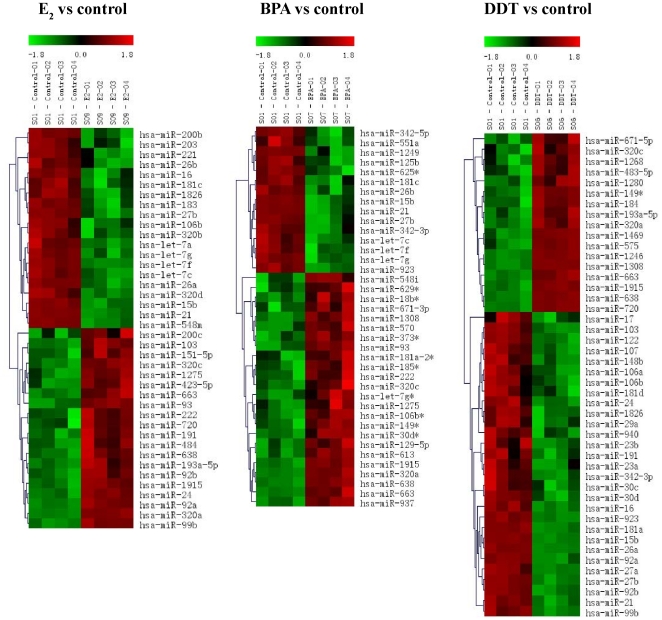
MicroRNA microarray heatmaps of MCF-7 cells treated with vehicle, estrogen, BPA, or DDT. (A) Estrogen versus control (B) BPA versus control and (C) DDT versus control. Arrowheads represent expression of miR-21.

**Figure 4:**
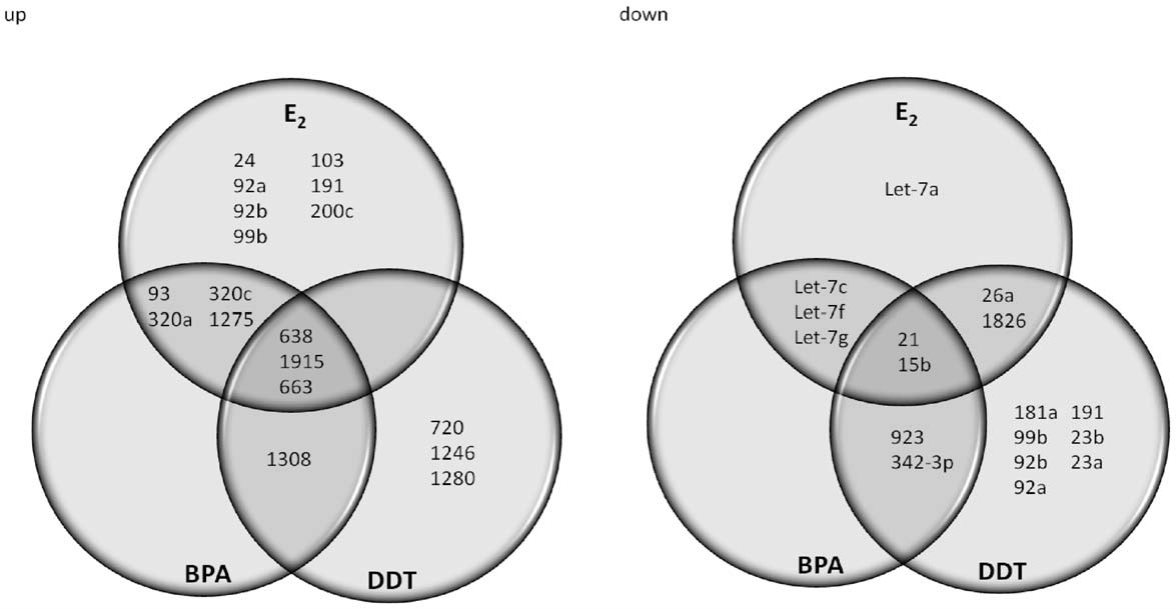
MicroRNA microarray of MCF-7 breast cancer cells treated with E_2_, BPA and DDT. Venn diagram showing the relative expression profiles of microRNA's from MCF-7 cells treated with estrogen vs BPA vs DDT.

**Figure 5:**
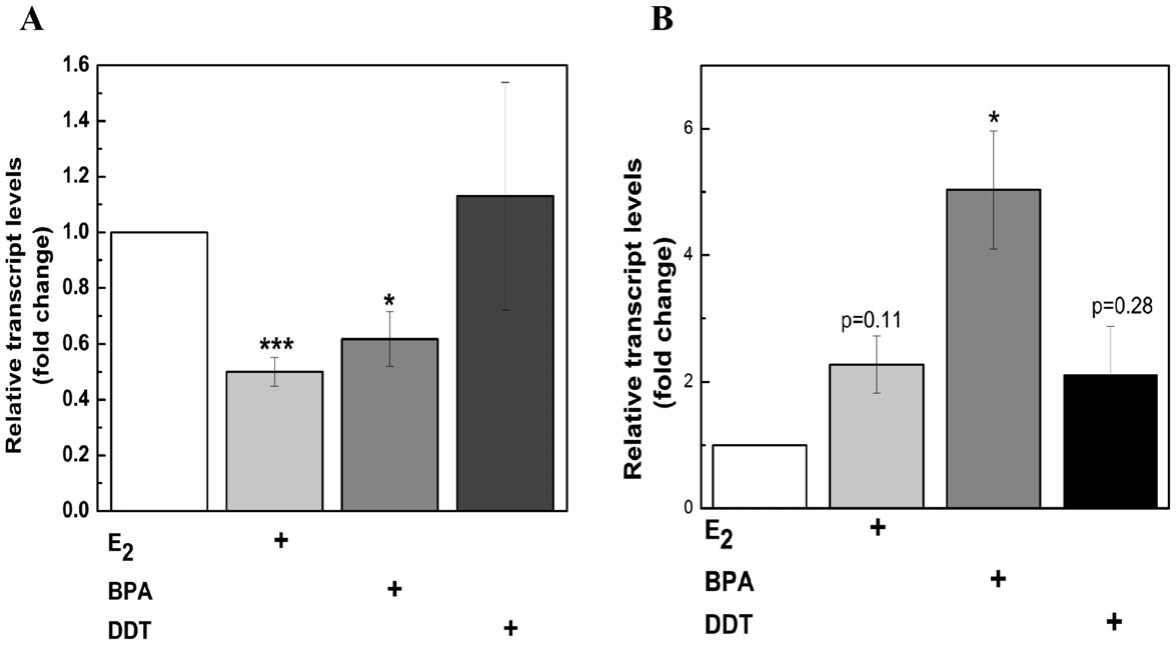
Effects of E_2_, BPA and DDT on miR-21 gene expression in MCF-7 and MCF-7F cells. MCF-7 cells (A) and MCF-7F cells (B) were treated with either vehicle, 1 nM E_2_, 10 µM BPA, or 10 µM DDT. Total microRNA was extracted followed by RT-PCR. * = p<0.05, ** = p<0.001.

Previous studies have shown that the onco-miR-21 is an estrogen-regulated microRNA and as such, plays an important role in breast cancer [25], [27], [35]. Based on the above results of our transcriptional activation assays and gene expression analyses, we investigated whether the EDCs could induce microRNA expression in hormone-responsive breast cancer cells. MCF-7 cells were exposed to vehicle, 10 µM BPA, 10 µM DDT, or 1 nM E_2_ for 18 hours, followed by total RNA extraction and analysis using microRNA microarrays of all known human microRNA species identified by the Sanger miRbase. Heatmaps were generated to compare the microRNA expression between treatments and controls. As shown in Figure 3, a number of microRNAs were significantly (p<0.01) altered by E_2_, DDT, and BPA. Treatment with E_2_, BPA and DDT decreased (p<0.05) miR-21 expression with a log 2 (G2/G1) value of −3.78, −3.72 and −0.93 respectively (Table 1). Several members of the let-7 family (let-7a, let-7b, let-7c, let-7d, let-7e and let-7f), were downregulated (p<0.05) by all three treatments, consistent with published data in human breast cancer cells [36], and miR-15b (p<0.005) and miR-27b (p<0.01) were also downregulated by the three treatments (Table 2). In addition, upregulation of miR-638 (P<0.005), miR-663 (P<0.005), and miR-1915 (P<0.005) was observed after treatment of MCF7 cells with E_2_, BPA or DDT (Table 2). Results from the microarray revealed many microRNAs were altered; however, levels of only 95 microRNAs were significantly regulated by the treatments. Interestingly, only five microRNAs were significantly regulated by all three treatments as demonstrated by the Venn diagram (Figure 4). In addition, the miR-21 microarray results were validated in MCF-7 cells by qRT-PCR (Figure 5), with both estrogen and BPA significantly suppressing miR-21 expression. Although the DDT treated cells showed an overall trend of decreasing miR-21 expression, the results were not statistically significant. To test the involvement of ERα in the suppression of miR-21 by treatment with E_2_, BPA, or DDT, we utilized MCF-7F cells which are derived MCF-7 cells that are ERα-negative and estrogen-resistant [37]. We found that in an ER (−) breast line E_2_ was unable to significantly alter expression of miR-21 (Fig. 5B). Interestingly, in MCF-7F cells, BPA stimulated an increase in miR-21 expression, in contrast to the suppression of miR-21 levels observed in the ERα (+) MCF-7 parental cells. The differential regulation of miR-21 by BPA between the two cell lines potentially reflects a role for BPA in regulation of distinct ER-dependent and independent signaling mechanisms in these cell systems. In any case, our results collectively demonstrate for the first time the ability of the xenoestrogens BPA and DDT to regulate microRNA expression in breast cancer cells, including miR-21, an estrogen-regulated onco-miR in breast cancer.

**Table 2:** MicroRNA microarray of MCF-7 cells.

Genes	E2	BPA	DDT
miR-21	−3.78	−3.72	−0.93
miR-638	0.69	1.28	1.12
miR-663	1.03	1.72	1.17
miR-1915	1.21	1.70	1.43
let-7g	−2.04	−2.39	n/s
let-7c	−1.36	−0.79	n/s
miR-923	n/s	−0.32	−0.51
miR-93	1.02	0.71	n/s
miR-320a	1.28	0.96	n/s
miR-1308	n/s	0.92	1.63
let-7f	−1.85	−0.76	n/s
miR-15b	−1.18	−2.07	−2.16
miR-1275	0.71	0.64	n/s
miR-27b	−1.91	−1.55	−1.54
miR-222	0.99	1.19	n/s
miR-193a-5p	1.15	n/s	0.50
miR-16	−1.44	n/s	−0.71
miR-26b	−2.35	−3.34	n/s
miR-149	n/s	1.53	1.86
miR-92a	0.67	n/s	−1.17
miR-99b	0.87	n/s	−0.55
miR-92b	0.52	n/s	−1.13
miR-342-3p	n/s	−0.99	−0.65

MicroRNA microarray profile of MCF-7 cells treated with vehicle, E_2_, DDT, or BPA. Numbers indicate significant –fold changes in microRNA expression compared to vehicle.

### Response of miR-21 regulated genes to DDT and BPA exposure in MCF-7 cells

**Figure 6:**
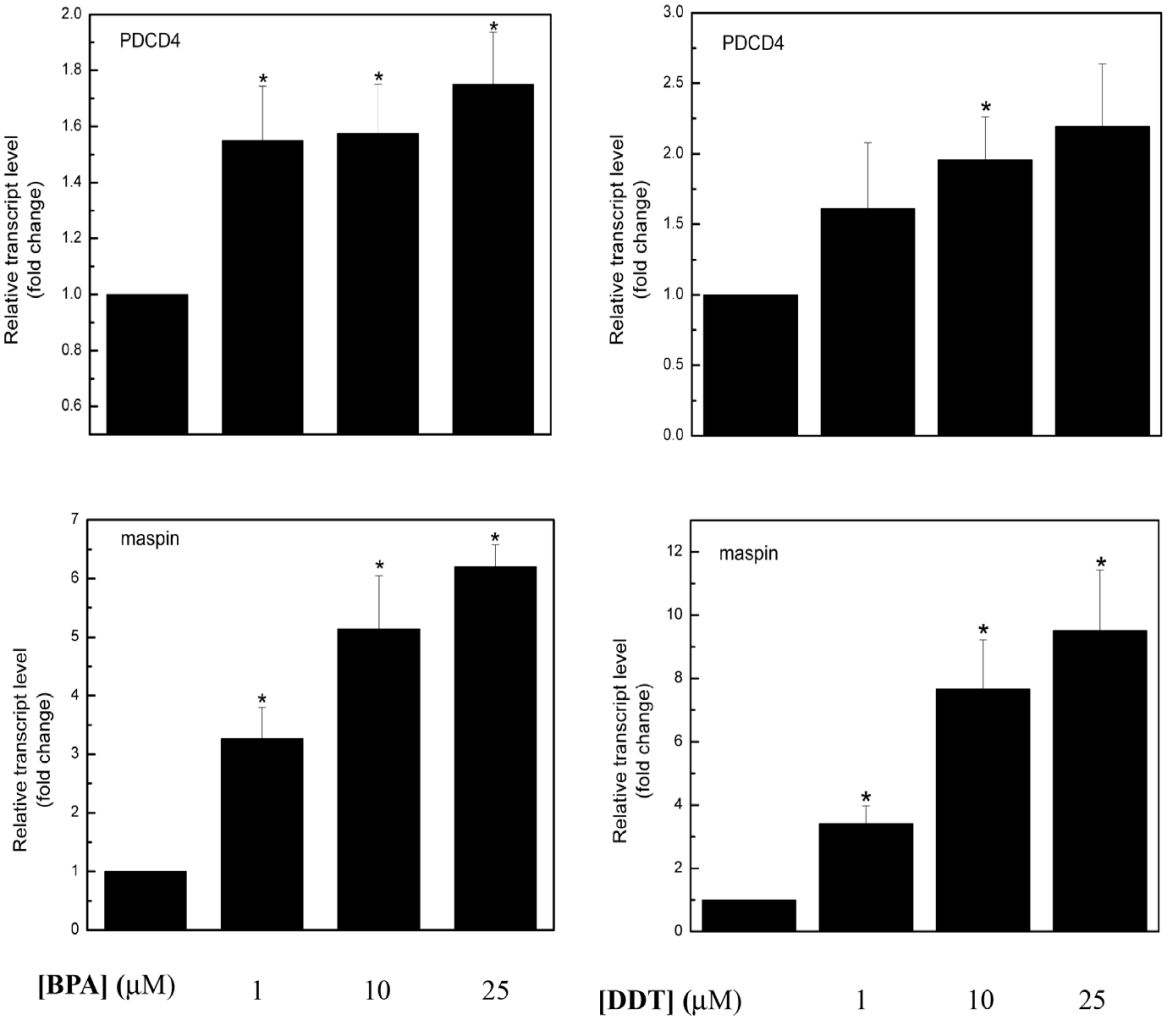
The effect of BPA and DDT on endogenous miR-21 target genes in MCF-7 cells. MCF-7 cells were treated for 18 hrs. with 0, 1, 10 and 25 µM BPA or DDT followed by RT-PCR of PDCD4 and maspin.

Previous studies have identified programmed cell death 4 (neoplastic transformation inhibitor) (PDCD4) and maspin as direct targets of miR-21 [38]. PDCD4 is a putative target of miR-21 and is implicated in the metastatic potential of pancreatic tumors [39], and maspin is a well-known tumor suppressor that can suppress tumor growth, invasion and metastasis [40]. Since E_2_, BPA and DDT reduced miR-21expression in MCF-7 cells, it was of interest to examine whether the effect of BPA and DDT resulted in altered expression of the two miR-21 target genes. MCF-7 cells were treated with increasing concentrations of BPA and DDT, and expression of maspin and PDCD4 was examined by RT-PCR. Results demonstrate a dose dependent increase in the expression of maspin and PDCD4 by BPA and DDT (Figure 6), suggesting that inhibition of miR-21 by DDT or BPA resulted in the increased expression of these two miR-21 target mRNAs. Interestingly, maspin appeared to be more sensitive to BPA and DDT than PDCD4 (Figure 6).

## Discussion

Understanding the cellular response to environmental endocrine disrupting chemicals is critical to defining the role of these agents in human health. We and others have shown the estrogenic potential of the plasticizer BPA and the insecticide DDT [41], [42]; however, the underlying mechanisms by which these compounds exert their effects and the biological endpoints that accompany them are not fully understood. In this study, we compared the estrogenic activity of BPA and DDT to that of the natural hormone estrogen in the ER (+) breast cancer cell line MCF-7. By using ERE-luciferase assays and quantitative PCR techniques, we were able to show BPA and DDT influence estrogen receptor activity at the transcriptional level in a manner similar to E_2_ (Figures 1 and 2, Table 1). Although our PCR superarray data revealed many genes were either up- or down-regulated by all three compounds, the altered expression of several genes by either DDT or BPA was observed, including Fas and Jun (Table 1).

Based on the differential mRNA expression patterns induced by BPA and DDT, we hypothesized that these two endocrine disruptors may induce distinct microRNA profiles in MCF-7 cells. The analysis of estrogen responsive microRNAs across several cell systems, including zebrafish [43], rat breast [44], human breast [45], mouse uterus [46], human myometrium and leiomyoma [23], and endometrium [47] has revealed patterns of estrogen-induced microRNA expression; however, the effect of environmental estrogens on microRNA expression in breast cancer cells has not been determined. We observed both common and distinct microRNA expression patterns in estrogen-treated MCF-7 cells. For example, E_2_ caused a down-regulation of miR-21 in MCF-7 cells, as seen by others [17], [45], while a unique E_2_-mediated decrease in miR-27b was observed. Our data show a complex, differential expression pattern of microRNA families induced by each individual compound: estrogen, BPA, and DDT each altered unique sets of microRNAs (Figure 4), strongly supporting our hypothesis that the activity of these two known xenoestrogens differs from the naturally occurring steroid hormone.

In breast cancer biopsies, increased expression of mir-21 has recently been reported [48]. Our microRNA array results suggest that in addition to E_2_, BPA and DDT affect endogenous levels of miR-21 in MCF-7 breast cancer cells. We first confirmed the microRNA array data showing E_2_, DDT, and BPA all downregulated miR-21 expression, although consistent with the array, DDT was not as effective as E_2_ or BPA (Figure 5). The mRNAs encoding maspin and PDCD4 are known targets of miR-21. We therefore confirmed that both BPA and DDT, in a dose-dependent manner, increased the transcript levels of each protein, consistent with a BPA- and DDT-mediated decrease in miR-21 levels (Figure 6). Recently, miR-21 was identified as being up-regulated by E_2_ in MCF-7p cells, a derivative of MCF-7 cells containing a bicistronic vector [16]. We can only speculate that the differences seen in our report are due to slight variations in cell lines (we used MCF-7 parental cells) or a difference in the amount of time cells were exposed to the drugs. However, an E_2_-mediated decrease in miR-21 was also reported by two groups using parental MCF-7 cells [17], [45]. Because elevated levels of miR-21 have been reported in breast tumor biopsies, an E_2_-mediated decrease in miR-21 expression might seem counterintuitive. However, the complex nature of breast tumor biology *in vivo* differs markedly from *in vitro* cultured MCF-7 primary breast cancer cells, and our results further indicate that microRNA expression in breast cancer cells undergo dynamic changes as cells transition from a primary, non-metastatic state (MCF-7) to that of an aggressive breast tumor. In addition, our results in the ERα (−) MCF-7F cells showing BPA stimulated an increase in miR-21 suggests an intriguing alternate mechanism by which BPA may exert both ERα-dependent and independent effects on regulation of coding and non-coding RNA levels (Fig. 5B). This observation further supports emerging evidence that EDCs such as DDT and BPA can influence multiple signaling cascades and gene expression networks in distinct cellular systems. Finally, our data are also the first to show an effect of EDCs on microRNA expression levels in a manner both similar and distinct from those seen by E_2._

A role for global microRNA changes in response to environmental signaling such as hypoxia, heavy metals, air pollution and dioxins is supported by a number of recent studies [49], [50]. The commercial use of BPA is increasing in frequency and exposure to BPA, through plastic beverage containers, has been shown to have significant deleterious effects on the endocrine system [51]. Likewise, atmospheric deposition of DDT remains a critical contaminant in the environment that has harmful effects on the liver, reproductive system, nervous system and ultimately can lead to cancer [52]. We show for the first time that both BPA and DDT can elicit changes in microRNA expression in human breast cancer cells, including oncomiR-21, supporting the possibility that environmental compounds with estrogenic activity have the potential to play an important role in breast carcinogenesis. Defining the molecular mechanisms underlying EDC-induced microRNA changes and the subsequent cellular consequences may contribute to the emerging roles of microRNAs in human health and disease.

## Materials and Methods

### Chemicals

4-Hydroxytamoxifen and BPA were purchased from Sigma-Aldrich (St. Louis, MO). Fulvestrant (ICI 182,780) was purchased from Tocris Bioscience (Ellisville, Missouri). o,p′ DDT was purchased from AccuStandard (New Haven, CT).

### Cell Culture

Human MCF-7 breast cancer cells were purchased from American Type Culture Collection (Manassas, VA) and were cultured in 75 cm^2^ culture flasks in DMEM (Invitrogen, Co.) supplemented with 5% FBS (Life Technologies, Inc., Gaithersburg, MD), basic minimum MEM essential (50×, Invitrogen Co.) and MEM non-essential (100×, Invitrogen, Co.) amino acids, sodium pyruvate (100× Invitrogen Co.), antimycotic-antibiotic (10,000 U/mL penicillin G sodium; 10,000 µg/mL streptomycin sulphate; 25 µg/mL amphotericin B as Fungizone®). The culture flasks were maintained in a tissue culture incubator in a humidified atmosphere of 5% CO_2_ and 95% air at 37°C. For estrogen studies, cells were washed with PBS three times and grown in phenol red-free DMEM supplemented with 5% dextran-coated charcoal-treated FBS (5% CS-FBS) for 48 h before plating for each particular experiment.

### Luciferase Assays

MCF-7 cells were seeded at 50–60% confluency in 24-well plates in DMEM containing 5% charcoal stripped fetal bovine serum (CS-FBS). Twenty-four hours later, each well was transfected with 0.2 µg of ERE-luciferase plasmid (Panomics) using Effectene (Qiagen) transfection reagent. Briefly, plasmid DNA was mixed with EC buffer (75 mL/mg DNA), followed by the addition of enhancer solution (3 mL/mg DNA). Samples were incubated at room temperature for 5 minutes followed by addition of the effectene reagent (6 uL/ug DNA). Samples were mixed and incubated at room temperature for 10 minutes. The DNA was then added to the cells. Four hours post transfection, either vehicle (DMSO) or drug (estrogen, o,p′-DDT, or BPA) was added. Cell lysates were harvested 18–24 hours later using MPER extraction reagent (Pierce) and read in a Berthold luminometer using Bright Glo luciferase assay reagent (Promega). Transfection efficiency was normalized by total protein concentration of each sample using the BCA assay (Pierce). Relative luminescence was calculated by dividing raw luminescence values by total protein, and each value is an average of three independent samples.

### Gene SuperArrays

MCF-7 cells were maintained in 10% FBS without phenol red. MCF-7 cells were withdrawn from estrogen in 10% CS-FBS for 6 days prior to plating for treatment. Cells were seeded into 6-well plates at 4×10^5^ cells/mL in 2.5 mL of 10% CS-FBS media per well the day before treatment. On the following day cells were treated with either vehicle, 1 nM E_2_, 10 µM BPA, or 10 µM o,p′-DDT for 48 hours. Total RNA was extracted and quality was checked with Experion (BioRad). Each array profiles the expression of a panel of 96 genes. For each array (n = 3), 4 µg RNA was reverse transcribed into cDNA using the First Strand Synthesis kit (SABiosciences) in the presence of gene-specific oligonucleotide primers as described in the manufacturer's protocol. cDNA template was mixed with the appropriate ready-to-use PCR master mix, equal volumes were aliquoted to each well of the same plate, and a real-time PCR cycling program was run. Quantitative RT-PCR was performed using manufacturer's protocols for the RT^2^ Profiler™ PCR Array (Human Breast Cancer and Estrogen Receptor Signaling Superarray, Gaithersburg, MD, USA). Relative gene expressions were calculated using the 2^−ΔΔCt^ method, in which Ct indicates the fractional cycle number where the fluorescent signal reaches detection threshold. The ‘delta–delta’ method uses the normalized ΔC_t_ value of each sample, calculated using a total of five endogenous control genes (18S rRNA, HPRT1, RPL13A, GAPDH, and ACTB) [53]. Fold change values are then presented as average fold change = 2^−^(^average ΔΔCt^) for genes in treated relative to control samples. Clinical variables were characterized using descriptive statistics, and the statistical significance of differences in gene expression between groups was calculated using the student's t-test.

### Real Time RT-PCR

Total cellular RNA was extracted using the RNeasy® mini column (Qiagen), following the manufacturer's instructions. Reverse transcription (RT) was carried out using the SuperScript First-Strand Synthesis System for RT-PCR (Invitrogen). One microgram of total RNA was reverse transcribed to cDNA following the manufacturer's instructions. For each RT, a blank was prepared using all the reagents except the RNA sample (for which an equivalent volume of DEPC treated water was substituted). This blank was used as a non-template control in the real-time PCR experiments. The level of SERPINB5 (maspin) and PDCD4 transcripts were determined using quantitative real-time PCR. Primers for PCR were designed to span intron/exon junctions to minimize amplification of residual genomic DNA. The primer sequences for SERPINB5 and PDCD4 are (sense and anti-sense, respectively): SERPINB5 (5′- CCTGTTCCTTTTCCACGCATTTTC-3′; 5′- CACCTTTAGCACCCACTTGAGC- -3′) and PDCD4 (5′-ATTTCAGCATCCTCCATTAACG-3′; 5′- GCCTATCCAGCAACCTTCC -3′), PCR mix contained optimal concentrations of primers, cDNA and SYBR Green PCR Master Mix (Bio-Rad Lab). Quantification and relative gene expression were calculated with internal controls with an n = 3. The ratio between these values provided the relative gene expression levels.

### MicroRNA Real Time RT PCR

MCF-7 cells were grown in 5% CS-FBS DMEM media for 48 hours prior to treatment with 100 pM 17-beta estrogen, 10 µm BPA, 10 µm op'DDT, or DMSO for 18 hours. Cells were harvested and total RNA extraction was performed using Qiagen miRNeasy RNA purification system according to the manufacturer's protocol. The quantity and quality of the total RNA was determined by measuring the absorbance at 260 and 280 nm using the NanoDrop ND-1000. 1.5 ug of total RNA was reverse-transcribed using the Qiagen Reverse Transcription kit as per manufacturer's protocol. qPCR was performed using SYBR green (Biorad) and primer for mature miR-21 (SAB Biosciences) as per manufacturer's protocol. Data was analyzed by comparing relative microRNA expression to U6 RNA. Relative microRNA expression was analyzed using 2-ΔΔCt method.

### microRNA Microarray Analysis

The cells were treated for 18 hours with vehicle, E_2_, DDT or BPA (n = 3). microRNA was extracted using the miRNeasy® mini column (Qiagen), following the manufacturer's instructions. Microarray assay was performed using a service provider (LC Sciences). The company used 2 to 5 µg total RNA sample, which was size fractionated using a YM-100 Microcon centrifugal filter (from Millipore) and the small RNAs (<300 nt) isolated were 3′-extended with a poly(A) tail using poly(A) polymerase. An oligonucleotide tag was then ligated to the poly(A) tail for later fluorescent dye staining; two different tags were used for the two RNA samples in dual-sample experiments. Hybridization was performed overnight on a μParaflo microfluidic chip using a micro-circulation pump (Atactic Technologies) [54]. On the microfluidic chip, each detection probe consisted of a chemically modified nucleotide coding segment complementary to target microRNA (from miRBase, http://microrna.sanger.ac.uk/sequences/) or other RNA (control or customer defined sequences) and a spacer segment of polyethylene glycol to extend the coding segment away from the substrate. The detection probes were made by *in situ* synthesis using PGR (photogenerated reagent) chemistry. The hybridization melting temperatures were balanced by chemical modifications of the detection probes. Hybridization used 100 µL 6xSSPE buffer (0.90 M NaCl, 60 mM Na_2_HPO_4_, 6 mM EDTA, pH 6.8) containing 25% formamide at 34°C. After hybridization detection used fluorescence labeling using tag-specific Cy3 and Cy5 dyes. Hybridization images were collected using a laser scanner (GenePix 4000B, Molecular Device) and digitized using Array-Pro image analysis software (Media Cybernetics). Data were analyzed by first subtracting the background and then normalizing the signals using a LOWESS filter Locally-weighted Regression [55]. A total of 4 technical replicates were used. For two color experiments, the ratio of the two sets of detected signals (log2 transformed, balanced) and p-values of the t-test were calculated; differentially detected signals were those with less than 0.01 p-values.
